# Impulse phobias during pregnancy: a case report of a 37 year-old woman pregnant of her first child

**DOI:** 10.1192/j.eurpsy.2024.1669

**Published:** 2024-08-27

**Authors:** M. Ríos-Vaquero, G. Lorenzo-Chapatte, L. Rojas-Vázquez, A. Monllor-Lazarraga, L. Sobrino-Conde, M. J. Mateos-Sexmero, T. Jimenez-Aparicio, M. Calvo-Valcarcel, M. A. Andreo-Vidal, M. P. Pando-Fernández, P. Martínez-Gimeno, M. D. L. A. Guillen-Soto, B. Rodriguez-Rodriguez, N. Navarro-Barriga, M. Fernández-Lozano, A. Aparicio-Parras, M. D. C. Vallecillo-Adame, C. De Andres-Lobo, A. Rodríguez-Campos

**Affiliations:** Psiquiatría, Hospital Clínico Universitario de Valladolid, Valladolid, Spain

## Abstract

**Introduction:**

Pregnancy and puerperium are two critical stages for women’s mental health due to the biological stress of pregnancy itself, as well as the emotional stress that surrounds this vital moment. (1) Debut and aggravation of psychiatric symptoms may occur, as well as relapse in women previously diagnosed with Severe Mental Disorder (SMD).

Symptoms of the anxious spectrum are the most frequent within the perinatal mental pathology, being impulse phobias an entity that appears in about 25% of women previously diagnosed with OCD and up to 10-15% of women without previous psychopathology (2)

**Objectives:**

Exposing the importance of Perinatal Mental Health from the presentation of a clinical case.

**Methods:**

Review of the literature available in PubMed. Presentation of the pathobiography and evolution of the patient.

**Results:**

Our case is about a 37-year-old woman, 30 weeks pregnant with her first child and history of having required admission to Psychiatry with subsequent follow-up in Mental Health for anxious-depressive symptoms with the presence of self-injurious ideas who, after two weeks with multiple life stressors, came to the Emergency Department for the presence of impulse phobias focused on pregnancy with significant internal anguish and ideas of death as a resolution to it, which is why it was decided to hospitalize her. During admission, and taking into account the patient’s gestational state, treatment was started with diluted Mirtazapine and Aripiprazole solution at minimal doses, which in this case were sufficient for symptom control.

The latest guidelines addressing psychopharmacology during pregnancy and lactation point to sertraline among the antidepressants and Lorazepam among the benzodiazepines as the safest drugs during pregnancy (3).

**Conclusions:**

- The exacerbation of anxious symptomatology and the presence of gestation-focused impulse phobias are frequent during pregnancy and their intensity increases as the time of delivery approaches.
- Sertraline, Lorazepam, Mirtazapine and Aripiprazole are safe drugs during pregnancy.
- In these women, a close and multidisciplinary follow-up by Psychiatry and Gynecology is advisable.

**Disclosure of Interest:**

None Declared

